# A case of acute myeloid leukemia and multiple myeloma is observed to occur simultaneously

**DOI:** 10.1097/MD.0000000000045190

**Published:** 2025-10-24

**Authors:** Shanfeng Hao, Yingqi Li, Huaquan Wang

**Affiliations:** a Department of Hematology, Tianjin Medical University General Hospital, Tianjin, People’s Republic of China; b Department of Neurology, Characteristic Medical Center of Chinese People’s Armed Police Force, Tianjin, People’s Republic of China.

**Keywords:** acute myeloid leukemia, azacitidine, brain abscess, multiple myeloma

## Abstract

**Rationale:**

Cases of acute myeloid leukemia occurring simultaneously with multiple myelomas are rare. To date, only 31 cases have been reported worldwide. The prognosis of these patients is very poor, and it is very important for them to be diagnosed promptly and treated effectively.

**Patient concerns:**

A 68-year-old male patient was admitted due to the presence of intermittent nosebleeds accompanied by fever for 10 days, in addition to the development of a right neck mass over 3 days.

**Diagnoses:**

Diagnosis of acute monocytic leukemia (high-risk group) was based on morphology, immunophenotyping, histochemical staining, and chromosomal and genetic test results. The patient’s chromosomes were found to be normal, yet next-generation sequencing revealed a TP53 mutation, thus classifying the risk stratification as high risk. The diagnosis of multiple myeloma was diagnosed based on the presence of > 10% myeloma cells and bone marrow biopsy findings suggestive of multiple myeloma.

**Interventions:**

Azacitidine 100 mg subcutaneous injection on days 1 to 7, in conjunction with hydrocortisone 50 mg every 12 hours, to treat acute myeloid leukemia in conjunction with multiple myeloma. During this course of treatment, the patient was administered anti-infectious therapy.

**Outcomes:**

The patient developed a brain abscess during treatment and passed away 2 months after hospitalization.

**Lessons:**

The patient’s disease was severe and rapidly progressive, and comorbid severe infections and consistent comorbid severe pancytopenia posed challenges in managing this disease. It is hoped that more effective targeted therapies can be explored for such patients.

## 1. Introduction

Acute myeloid leukemia (AML) is a malignant hematological disease that originates in primitive hematopoietic cells. Multiple myeloma (MM) is a hematological malignancy characterized by uncontrolled proliferation of plasma cells that secrete monoclonal immunoglobulin and its fragments (M protein). This leads to damage to related tissues and organs. These 2 types of hematological malignancies have disparate cellular origins. AML arises from myeloid hematopoietic cells, whereas MM originates from plasma cells that differentiate into lymphoid hematopoietic cells. The rarity of this case lies in the fact that the patient was simultaneously diagnosed with 2 malignant tumors of different cellular origins. The patient presented with a dual diagnosis of AML and MM and had no history of radiotherapy or chemotherapy. This article presents a case report to remind hematologists to exercise caution and thoroughness in examination when confronted with critical and challenging cases, and to pursue novel therapeutic avenues for such rare and poor prognosis cases.

## 2. Case presentation

### 2.1. History and physical examination

The patient, a 68-year-old male, was admitted on March 24, 2020, due to the presence of intermittent nosebleeds accompanied by a fever for 10 days, in addition to the development of a right neck mass over 3 days. The patient’s medical history is as follows: Ten days prior to admission, the patient experienced a significant amount of nosebleed after trimming the nasal hair on the right nostril. The patient was admitted to the emergency department of our hospital and was found to have a fever with a maximum body temperature of 39.0°C, accompanied by sweating, and no other accompanying symptoms. A complete blood count was performed to assess the patient’s hematological parameters. White blood cell count: 2.21 × 10⁹/L (decreased), red blood cell count: 1.82 × 10¹²/L (decreased), hemoglobin: 69 g/L (decreased), platelet count: 25 × 10⁹/L (decreased), absolute neutrophil count: 0.37 × 10⁹/L (decreased).Chest computed tomography (CT) scan: increased interstitial texture and interstitial lesions in both lungs; low-density shadows in the liver. The patient was treated in an emergency setting, which included the administration of symptomatic measures such as hemostatic agents and antipyretic medications. The patient was discharged after the cessation of nosebleeds. Following their discharge from the hospital, the patient continued to experience intermittent fever, with a maximum body temperature of 39.0°C. The patient was then initiated on a self-cooling treatment regimen at home. Three days prior to admission, the patient presented with nasal bleeding, accompanied by fever, profuse sweating, and the appearance of a mass measuring approximately 4 cm in diameter on the right side of the neck. The mass was characterized by local redness, swelling, tenderness, and a slightly elevated temperature of the surrounding skin. Furthermore, the patient reported a cough without any other accompanying symptoms. The patient was subsequently evaluated in the emergency department of our medical center. An ultrasonographic examination of the superficial lymph nodes revealed an increase in size of the right submandibular gland in comparison to the opposite side, as well as thickening of the right sternocleidomastoid muscle. Furthermore, multiple hypoechoic nodules were observed in the right cervical region II-IV, except for the abnormally enlarged lymph nodes, for which further examination was recommended. A further thoracic CT scan was conducted for the purpose of enabling comparison with the previous scan. This revealed an increase in the density of the fat interstitial space in the anterior chest wall, subcutaneous tissue, and anterior mediastinum at the level of the thoracic inlet. Both lungs exhibited increased interstitial texture and thickening of some interlobular septa, as previously observed. No evidence of enlargement was observed in the lymph nodes of the mediastinum, as had been previously noted. A transfusion of platelets and red blood cells was administered to the patient with the objective of providing symptomatic support. The patient was admitted to our department for further diagnostic evaluation and treatment.

The patient was previously in good health, with no significant medical history. His wife succumbed to leukemia, and he had no children. Upon physical examination, the patient’s temperature was recorded at 37.8°C, with a pulse rate of 94 beats per minute, respiratory rate of 18 beats per minute, and blood pressure of 112/64 mm Hg (1 mm Hg = 0.133 kPa). The patient was conscious and was able to respond fluently. He was anemic, but did not exhibit any yellowing of the sclera, conjunctival congestion, lid conjunctival pallor, pale lips and mouth, or pharyngeal redness. Instead, he displayed scattered hemorrhagic dots on the anterior end of the tongue. No ulcers were observed on the oral mucosa. The right side of the neck was visible as a mass approximately 8 cm in diameter, with localized redness and swelling, tenderness, slightly elevated skin temperature, and no breakage of the skin surface. The thyroid gland was not enlarged, and the sternum did not exhibit compression. The respiratory sounds of the lungs were of thick quality, and no discernible dry or wet rales were audible. The heart and abdomen exhibited no abnormalities, and there was no edema in either lower extremity.

### 2.2. Examination and diagnosis

**Figure 1.**
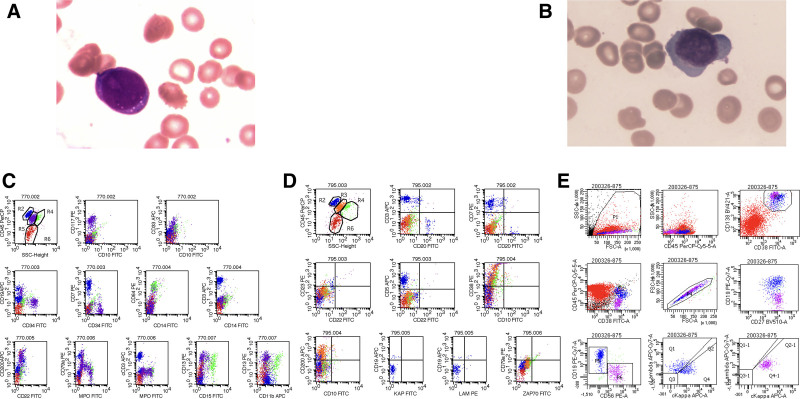
(A) Bone marrow morphology revealed monoblast. (B) Bone marrow morphology revealed myeloma cell. (C) The leukemia phenotype determined by flow cytometry exhibited a 42.26% primitive cell population that exhibited partial expression of CD117, CD34, CD64, CD33, HLA-DR, MPO, and CD13. (D) The lymphoid phenotype demonstrated the R3 phenotype exhibited 35.04% expression of CD200 and CD38. (E) Flow cytometry revealed 2.43% abnormal plasma cells with a particular profile: CD38+, CD138+, CD56+, CD27+, CD19-, CD45-, cKAP+, and cLAM-.

Routine blood examinations were performed following admission. White blood cell count was 0.60 × 10⁹/L, NEUT count of 0.37 × 10⁹/L, red blood cell count of 1.42 × 10¹²/L, hemoglobin concentration of 53 g/L, and platelet count of 45 × 10⁹/L. Bone marrow morphology revealed 23.5% monoblasts, 2.5% promonocytes, and 14% proplasmacytes(shown in Fig. 1A), indicative of a combination of AML subtype M5^[1]^ with an increased percentage of abnormal plasma cells(shown in Fig. 1B). Histochemical staining revealed 33% myeloperoxidase positivity, with a positivity index of 50; 86% nonspecific esterase positivity, with a positivity index of 140; 44% nonspecific esterase + NaF positivity, with a positivity index of 53; 100% naïve cell glycogen positivity, with a positivity index of 131; and PAS-positive reactant with purple granules in a scattered distribution. The leukemia phenotype, as determined by flow cytometry, exhibited a 42.26% primitive cell population that exhibited partial expression of CD117, CD34, CD64, CD33, HLA-DR, myeloperoxidase, and CD13 (Fig. 1C). The lymphoid phenotype demonstrated 25% expression of CD3, CD7, CD5, and CD38, whereas the R3 phenotype showed 35.04% expression of CD200 and CD38 (Fig. 1D). The chromosomal karyotype was as follows: the patient was identified as 46, XY [12]. MDS-FISH revealed the presence of D7S486/CSP7 [del(7q31)] at a frequency of 4% (within the normal range of <3.56%). The leukemia 43 gene screen yielded no abnormalities. The leukemia prognostic gene next-generation sequencing test indicated the presence of a TP53 mutation in the patient, with a mutation rate of 10.95%. Bone marrow biopsy revealed normal bone marrow cellularity, no lymphocytosis but plasmacytosis was seen. Immunohistochemical staining revealed a few positive myeloperoxidase machines, scattered positive lysozyme, scattered positive CD138, a few weakly positive CD117, scattered positive CD34, and positive CD61 megakaryocytes. However, the positive localization of CD117 and CD34 in myeloid or plasma cells could not be clearly defined. The CD20 marker exhibited minimal positivity, whereas the CD3 marker showed slight positivity.The light chain staining in the bone marrow was ҡ≫ג. Considering its clinical presentation, the diagnosis of multiple myeloma (MM) was considered probable. Serum protein electrophoresis indicated that the proportion of monoclonal globulin to total protein was 13.1%, corresponds to 8.8 g/L. Additionally, serum immunofixation electrophoresis provided suggestive evidence of an IgG (+) κ (+) status. Serum ҡ/ג FLC ratio was > 100. Flow cytometry revealed 2.43% abnormal plasma cells with the following profile: CD38+, CD138+, CD56+, CD27+, CD19 −, CD45 −, cKAP+, and cLAM − (Fig. 1E). IgH rearrangement was not detected, and TCR-δ rearrangement was identified.

Diagnosis and differential diagnosis:1. Acute monocytic leukemia (high-risk group): The diagnosis is unambiguous based on morphology, immunophenotyping, histochemical staining, chromosomal, and genetic test results. The patient’s chromosomes were found to be normal, yet next-generation sequencing revealed a TP53 mutation, thus classifying the risk stratification as high-risk.^[2]^2. The patient was diagnosed with multiple myeloma (IgG, ҡ-type) (DS stage III, ISS stage II). This diagnosis was based on the presence of > 10% myeloma cells and a bone marrow biopsy suggestive of MM. Additionally, the presence of M protein (IgG, ҡ-type) and a plasma cell phenotype, which is indicative of the presence of clonal plasma cells (restricted expression of the ҡ-light chain), supported the diagnosis of MM.^[3]^ However, a diagnosis of symptomatic MM also necessitates evidence of target organ damage, namely, CRAB-SLIM symptoms. In the present case, Serum ҡ/ג FLC ratio was > 100.The patient presented with anemia; however, acute leukemia can also manifest with this symptom. In addition, the patient did not exhibit hypercalcemia or renal insufficiency. However, owing to financial constraints, the patient was unable to undergo PET/CT for bone destruction and extramedullary infiltration. During hospitalization, head and chest CT and left knee X-rays were performed; however, they did not reveal any evidence of bone destruction in these regions. AML combined with multiple myeloma is clinically rare and the diagnosis of AML is clear. However, the presence of M protein combined with plasmacytosis requires differentiation from reactive plasmacytosis and monoclonal gammopathy of undetermined significance (MGUS). In cases of reactive plasmacytosis, the proportion of bone marrow plasma cells is typically <10%, exhibiting well-differentiated characteristics. In addition, immunoglobulins are generally polyclonal, with only a few monoclonal instances and a limited increase. The significance of MGUS is generally indicated by the presence of monoclonal plasma cells comprising <10% of the total plasma cell population. However, in this case, the proportion of bone marrow plasma cells was more than 10%, and they were immature plasma cells. Serum protein electrophoresis of the patient showed the presence of monoclonal immunoglobulin. The patient’s myeloma cells expressed cd38 + cd56 + cd19-, while the plasma cells of reactive plasmacytosis showed cd38 + cd56- cd19+.Following comprehensive discussion, the diagnosis of AML combined with symptomatic MM was confirmed.

### 2.3. Treatment and outcome

**Figure 2.**
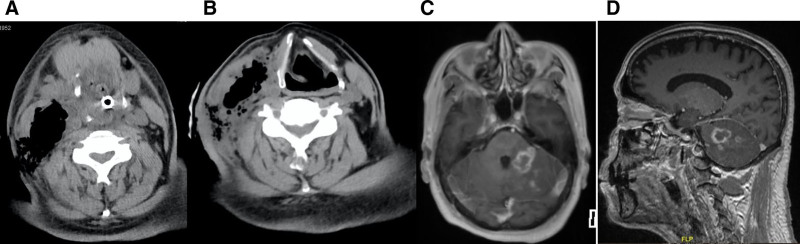
(A) The patient’s cervical CT on March 25 revealed multiple gas-dense shadows in the right cervical sternocleidomastoid muscle and subcutaneous soft tissues, with surrounding soft tissue swelling and blurring. (B) A repeat CT scan of the neck on April 3 revealed a slight improvement in the size of the neck mass and the severity of the soft tissue infection in comparison to the previous scan. (C) and (D) A head enhancement MRI conducted on April 20 revealed the presence of irregular signal shadows on the left side of the head, particularly in the left pontine arm and the left cerebellar hemisphere.

Upon admission, carbapenem antibiotics were administered for anti-infective treatment in accordance with the principles of granulomatous fever management. On the following morning, following defecation in the bathroom, the patient experienced syncope and loss of consciousness. The patient’s heart rate was 116 beats per minute, blood pressure was 136/71 mm Hg, and oxygen saturation progressively decreased to 33%. The patient was intubated via bedside endotracheal intubation and a substantial quantity of food residue was observed within the airway and epiglottis. The patient’s cervical CT revealed an enlarged “lymph node,” prompting transfer to the intensive care unit (ICU). Subsequent imaging revealed multiple gas-dense shadows in the right cervical sternocleidomastoid muscle and subcutaneous soft tissues, with surrounding soft tissue swelling and blurring (Fig. 2A). These findings suggested the presence of infectious lesions. Additionally, cranial CT showed increased density in the posterior cerebral falx, potentially indicating a subdural hematoma. The results of the blood culture were as follows: the isolate was identified as *Klebsiella pneumoniae*. The patient underwent ventilator-assisted respiration along with treatments aimed at improving cerebral metabolism, anti-infection, and blood transfusion. The patient also received other treatments in the ICU. Subsequently, the patient regained consciousness and was weaned off ventilator-assisted respiratory support. After a period of 3 days, the patient was transferred from the ICU to the hematology department for further treatment. A head CT scan indicated the possibility of a subdural hematoma; however, this was subsequently excluded using a head MRI scan. It was therefore postulated that the abrupt loss of consciousness was precipitated by a substantial neck mass in conjunction with an infection, which resulted in swelling of the soft tissues of the oropharynx and nasopharynx and narrowing and obstruction of the airway.

The patient was administered an azacitidine 100 mg subcutaneous injection on days 1 to 7 from April 2 to April 8, 2020, in conjunction with hydrocortisone 50 mg every 12 hours to treat AML in conjunction with MM. Azacitidine dose reduction was due to severe pancytopenia and serious infection. During this course of treatment, the patient was also provided with anti-infective therapy, transfusions of red blood cells and platelets, and erythropoietin and granulocyte colony-stimulating factor were administered to promote hematopoietic recovery. Ruyi Jinhuang San was applied topically to the infected area of the neck. Reexamination of bone marrow aspiration on May 14 showed that AML was not in remission and the number of plasma cells was lower than before. Monoclonal globulin decreased from 8.8 g/L to 2.09 g/L.The efficacy of AML was evaluated as primary drug resistance^[2]^and the efficacy of MM was evaluated as PR.^[4]^ A repeat CT scan of the neck was performed on April 3, 2020, which revealed a slight improvement in the size of the neck mass and severity of the soft tissue infection compared to the previous scan (Fig. 2B). During hospitalization, the patient exhibited an increasing inability to walk with stability, accompanied by a progressive worsening of the condition. Head enhancement MRI conducted on April 20, 2020, revealed the presence of irregular signal shadows on the left side of the head, particularly in the left pontine arm and left cerebellar hemisphere (Figs. 2C, 2D). This led to a diagnosis of an infectious lesion. Accordingly, the patient was diagnosed with a brain abscess and treated with a combination of anti-infective agents capable of traversing the blood-brain barrier. On May 15, 2020, the patient exhibited sudden slurred speech, crooked corners of the mouth, salivation, and choking on water. Urgent head CT was highly suggestive of cerebral infarction. Given the patient’s history of cerebral abscesses and sepsis, the possibility of cerebral abscess dissemination cannot be excluded. The patient’s family opted to discontinue treatment because of economic constraints. The patient died of the illness on May 20, 2020.

## 3. Discussion and conclusion

The patient presented with an acute onset of illness. The patient experienced loss of consciousness and hypoxemia prior to diagnosis. This was due to a large neck mass, severe infection, and airway stenosis. The patient was successfully treated promptly. The diagnosis of AML-M5 was confirmed through follow-up examinations. Regarding the etiology of plasmacytosis and M protein levels, we postulated that the patient had a severe infection secondary to plasmacytosis, which resulted in the production of monoclonal gamma globulin. Additionally, we considered the possibility that the patient’s neck mass was extramedullary plasmacytoma. However, the patient’s neck CT scan confirmed that the neck mass was a soft tissue infection of the neck, in conjunction with the patient’s bone marrow plasma cell monoclonal antibody and the proportion of monoclonal plasma cells exceeding 10%, leading to the conclusion that the patient met the diagnostic criteria for MM through the integration of various examinations. Therefore, this is an uncommon case of dual hematological malignancy.

A review of the literature from 1972 to 2024 identified 31 patients with AML and MM.^[5–11]^ Most cases involved patients aged > 60 years (23/31),^[5,8–10]^ including 21 males^[5–7,9]^and 10 females.^[8–10]^ Most patients had the IgG/κ or IgG/λ monoclonal globulin subtypes (15/28).^[5,9]^ Most patients reported in the early literature lacked the genetic molecular features. In contrast, 11 patients reported after 2000 underwent molecular genetic testing, which revealed a range of genetic features, including RB1, TP53, JAK2 V617F, TET2, NRAS, CEBPA double mutation, IDH2, NPM1, and DNMT3A.^[7–10]^ Notably, 4 patients had a TP53 mutation or a 17p deletion.^[7,9]^ Treatment strategies vary across the literature, but most tend to focus on AML treatment first, with “7 + 3” regimens, MP regimens, and other regimens dominating the early literature.^[9]^ Some patients were also treated with “7 + 3,” CAG, FLAG, or other regimens in combination with bortezomib.^[8,9]^ Recent studies have reported patients treated with azacitidine in combination with bortezomib, lenalidomide, and daratumumab.^[9]^ Despite the disparate therapeutic strategies, the literature indicates that most of these patients have a dismal prognosis, with only 9 patients surviving for over a year (9/27).^[7–10]^ Of these 9 patients, 2 exhibited prolonged survival after allogeneic hematopoietic stem cell transplantation following disease relapse.^[7,9]^ However, only 8 of these patients were < 60 years old, and most lacked access to allogeneic hematopoietic stem cell transplantation. Thus, selection of an optimal therapeutic regimen for this rare disease remains a significant challenge, particularly given the limited availability of stem cell transplantation options.

A specific analysis of 9 patients with survival exceeding 1 year revealed that, except for 1 patient who received azacitidine exclusively,^[9]^ the chemotherapy regimens of the remaining 8 patients encompassed both AML and MM. This included one patient with AML-M3 concomitant with MM, whose AML chemotherapy regimen entailed all-trans retinoic acid^[7]^; one patient with AML-M2 (presenting with an IDH2 mutation) in conjunction with MM, whose AML chemotherapy regimen was enasidenib^[9]^; and one patient who had an AML chemotherapy regimen of mitoxantrone plus cytarabine.^[9]^ The remaining 5 patients received chemotherapy regimens that included either azacitidine or low dose decitabine.^[8–10]^ This indicates that 6 out of 9 patients used demethylating agents, and 4 of these 6 patients had DNMT3A mutations.^[8–10]^ Azacitidine was also administered to one of the 18 patients with a survival period of less than 1 year.^[11]^ Similar to the patient in the present case, the patient had a severe skin infection at the onset of the disease and was treated with a chemotherapy regimen of azacitidine and bortezomib in combination with dexamethasone. The patient survived for 120 days after disease onset.^[11]^ The patient in our case also had a severe and uncontrollable soft tissue infection of the skin of the neck at the onset of the disease, which progressed to a brain abscess, and died soon afterward, surviving for less than 2 months, despite the use of chemotherapy with azacitidine in combination with glucocorticoids.

Furthermore, the survival prognosis of 14 patients with MDS-EB combined with MGUS/MM has been summarized previously summarized.^[12]^ The median age of the 14 patients was 65.5 years. Most participants (85.7%) exhibited severe anemia or pancytopenia and approximately half (42.9%) progressed to AML. Patients with MGUS/MM and MDS-EB exhibit markedly poor therapeutic responses. A significant proportion of the patients (64%) demonstrated no response or rapid relapse. The median overall survival for patients with MGUS/MM and MDS-EB was only 8 months, which was significantly shorter than that observed in patients with MGUS/MM and MDS-EB. The median overall survival for patients with MGUS/MM and MDS-other types was 52 months (*P* = .0009). These findings indicate that the prognosis of patients with concurrent myeloid and plasma cell tumors is poor.

The case report indicated that the patient presented with acute onset of illness and severe infection, which did not allow additional time to treat the disease. The combination of TP53 mutations also indicated an unfavorable prognosis. From the entirety of this case, it can be surmised that the patient exhibited severe disease onset, rapid progression, and a poor response to treatment, which aligns with the findings reported in the literature. This patient’s leukemia cells expressed CD38 by flow cytometry, and the expression level was as high as 92%. A study showed that in relapsed refractory AML patients with high CD38 expression, the complete remission rate with daratumumab could be as high as 57.1%.^[13]^ Daratumumab was a potentially better first-line treatment option for this patient, who had both AML and MM; however, the patient declined the drug at that time for financial reasons. The overall prognosis for this group of patients is unfavorable, which motivated us to pursue a more efficacious program to enhance the outcomes in this cohort.

## Author contributions

**Data curation:** Yingqi Li.

**Writing – original draft:** Shanfeng Hao.

**Writing – review & editing:** Huaquan Wang.
